# Seed dormancy cycling in Arabidopsis: chromatin remodelling and regulation of DOG1 in response to seasonal environmental signals

**DOI:** 10.1111/tpj.12735

**Published:** 2014-12-26

**Authors:** Steven Footitt, Kerstin Müller, Allison R Kermode, William E Finch-Savage

**Affiliations:** 1 School of Life Sciences, Wellesbourne Campus, University of WarwickWarwickshire, CV35 9EF, UK; 2 Biological Sciences, Simon Fraser University8888 University Dr., Burnaby, BC, V5A 1S6, Canada

**Keywords:** dormancy cycling, chromatin remodelling, histone ubiquitination, histone acetylation, histone methylation, Arabidopsis

## Abstract

The involvement of chromatin remodelling in dormancy cycling in the soil seed bank (SSB) is poorly understood. Natural variation between the winter and summer annual Arabidopsis ecotypes Cvi and Bur was exploited to investigate the expression of genes involved in chromatin remodelling via histone 2B (H2B) ubiquitination/de-ubiquitination and histone acetylation/deacetylation, the repressive histone methyl transferases *CURLY LEAF* (*CLF*) and *SWINGER* (*SWN*), and the gene silencing repressor *ROS1* (REPRESSOR OF SILENCING1) and promoter of silencing *KYP/SUVH4* (*KRYPTONITE*), during dormancy cycling in the SSB. *ROS1* expression was positively correlated with dormancy while the reverse was observed for *CLF* and *KYP/SUVH4*. We propose ROS1 dependent repression of silencing and a sequential requirement of CLF and KYP/SUVH4 dependent gene repression and silencing for the maintenance and suppression of dormancy during dormancy cycling. Seasonal expression of H2B modifying genes was correlated negatively with temperature and positively with *DOG1* expression, as were histone acetyltransferase genes, with histone deacetylases positively correlated with temperature. Changes in the histone marks H3K4me3 and H3K27me3 were seen on *DOG1* (*DELAY OF GERMINATION1*) in Cvi during dormancy cycling. H3K4me3 activating marks remained stable along *DOG1*. During relief of dormancy, H3K27me3 repressive marks slowly accumulated and accelerated on exposure to light completing dormancy loss. We propose that these marks on *DOG1* serve as a thermal sensing mechanism during dormancy cycling in preparation for light repression of dormancy. Overall, chromatin remodelling plays a vital role in temporal sensing through regulation of gene expression.

## Introduction

Seeds in the soil seed bank (SSB) continually adjust their dormancy status to synchronise germination and seedling emergence to an appropriate climate space and time of the year. This allows multiple species to compete successfully within species rich natural communities (Baskin and Baskin, 2006; Walck *et al*., 2011). Seeds sense and integrate a range of environmental signals to adjust their depth of dormancy. Some signals (e.g., soil temperature and moisture) are related to temporal sensing, the slow seasonal change that indicates when a suitable time of year exists (temporal window). Integrated over time these signals alter the depth of dormancy and the sensitivity to a second set of signals (e.g., light, nitrate, alternating temperatures). These second sets of signals are related to spatial sensing and indicate more immediately if conditions are suitable to terminate dormancy and induce the completion of germination (spatial window). This involves sensing soil depth (amplitude of diurnal temperature fluctuation; oxygen, water), soil disturbance (light; oxygen), and vegetation gaps (nitrate; light quality; the degree of diurnal temperature fluctuation) (Baskin and Baskin, 2006; Finch-Savage and Leubner-Metzger, 2006; Footitt *et al*., 2011, 2013, 2014; Finch-Savage and Footitt, 2012). If the correct spatial window does not occur, the temporal window will close for another year. These sensing and signalling mechanisms make seeds highly efficient in exploiting distinct habitats and climate spaces (Pons, 1989; Saatkamp *et al*., 2011a,b; Walck *et al*., 2011). The resulting dormancy cycling coupled with seed longevity represents a bet-hedging strategy for the short- and long-term persistence of native/weed species within the SSB (Evans and Dennehy, 2005; Walck *et al*., 2011; Footitt *et al*., 2014).

Mutant screening for altered dormancy and germination has been widely used to understand the genetic regulation of dormancy. Many, but not all, were found to be involved in the abscisic acid (ABA) and gibberellin (GA) metabolism and signalling pathways (Nambara *et al*., 2010; Graeber *et al*., 2012). This confirmed the central involvement of the ABA/GA balance hypothesis in the seed’s ability to interpret the environment thereby regulating dormancy and germination (Kucera *et al*., 2005; Finch-Savage and Leubner-Metzger, 2006), but upstream regulation especially during seed dormancy cycling in the SSB is little understood. In plants, adaptation to a changing environment is achieved by controlling gene expression at the genome level in part via changes in chromatin structure associated with histone modification (Chinnusamy and Zhu, 2009). In seeds, a screen for low dormancy mutants led to the identification of *HISTONE UBIQUITINATION1* (*HUB1*) encoding an E3 ligase with homology to the histone-modifying enzymes Bre1 (yeast) and RNF20 ⁄RNF40 (human) (Liu *et al*., 2007).

HUB1 is required for histone H2B monoubiquitination at Lys-143 (H2BK143) a prerequisite for histone H3 methylation at Lys-4 (H3K4me3), and Lys-79 (H3K79me3), both associated with gene activation (Du, 2012). In *Arabidopsis thaliana* this involves the genes *HUB1* and *2* and three genes encoding ubiquitin carriers *UBC1, -2, and -3 (UBIQUITIN CARRIER 1, -2, -3*) (Sun and Allis, 2002; Cao *et al*., 2008). HUB1 along with conjugating enzymes UBC1 and UBC2 combine to monoubiquitinate histone H2B (H2Bub1). In yeast, H2Bub1 is required for binding histone H3 lysine methylases to chromatin during the di- and tri-methylation of histone H3 at lysines 4 and 36 (H3K4 and H3K36) leading to transcriptional activation (Cao *et al*., 2008; Wright *et al*., 2011; Du, 2012). De-ubiquitination of H2Bub1 in Arabidopsis results in gene repression and silencing. In Arabidopsis the Otubain-like deubiquitinase, OTLD1 (OTUBAIN-LIKE HISTONE DEUBIQUITINASE) acts in a complex with the histone lysine demethylase, KDM1C to deubiquitinate H2Bub1 and demethylate H3K4 leading to gene repression (Krichevsky *et al*., 2011). Additionally, histone de-ubiquitination by the action of UBP26 (UBIQUITIN-SPECIFIC PROTEASE 26) maintains the H3K9 dimethylation induced by the histone methyltransferase KYP⁄SUVH4 (KRYPTONITE), a negative regulator of dormancy, leading to DNA methylation resulting in gene silencing in heterochromatin (Jackson *et al*., 2002, 2004; Sridhar *et al*., 2007; Zheng *et al*., 2012). DNA methylation is removed by DNA glycosylase enzymes such as ROS1 (REPRESSOR OF SILENCING1), which is itself important for H2B ubiquitination (Agius *et al*., 2006; Sridhar *et al*., 2007; Furner and Matzke, 2011).

Gene repression and silencing also involves the Polycomb repressive complexes (PRC) PRC1 and PRC2, and ubiquitination of histone 2A (H2A) at promoters of polycomb group targeted silenced genes. PRC2, contains the histone methyl transferases SWN (SWINGER) and CLF (CURLY LEAF) which methylate histone 3 lysine 27 (H3K27me3) resulting in gene repression. PRC1 interacts with H3K27me3 enabling it to conduct H2A monoubiquitination leading to chromatin compaction and repression of transcription (Du, 2012; Engelhorn *et al*., 2014; Molitor *et al*., 2014). In plants this also occurs in the reverse order with H2A ubiquitination followed by maintenance of repression by PRC2. However, PRC1 can also induce chromatin compaction in the absence of H2A ubiquitination. The order and involvement or not of H2A ubiquitination appears to depend on the target genes (Engelhorn *et al*., 2014). This flexibility in the PRC system potentially enables an appropriate balance of repressive marks (Yang *et al*., 2013; Calonje, 2014).

Gene activation and repression are also modulated by histone acetylation and deacetylation (Berr *et al*., 2011). Acetylation neutralises positively charged histone lysine groups reducing interaction between histones and DNA effectively relaxing the heterochromatin leading to gene activation. The balance of histone acetylation is maintained by antagonism between the histone acyltransferases (HATs) and deacetylases (Berr *et al*., 2011). In publically available seed dormancy microarray data (Cadman *et al*., 2006; Finch-Savage *et al*., 2007), histone acyltransferases and deacetylases exhibit expression patterns that change with dormancy. Of these, two members of each group with distinct expression patterns in dormancy microarrays were chosen for analysis: genes encoding the histone acyltransferases *ELO3* (*ELONGATA3*), a Gcn5-related *N*-acetyltransferases family member of the Elongator complex, and *HAC1* (*HISTONE ACYLTRANSFERASE1*), a CREB-binding protein family member; and the histone deacetylases *HD2A* (*HISTONE DEACETYLASE 2A*) of the HD2-like family and *HDA2* a member of the RPD3 deacetylase family (Berr *et al*., 2011). The HD2-like family are plant specific deacetylases, having specific roles in seeds and seedling growth (Berr *et al*., 2011; Colville *et al*., 2011; Yano *et al*., 2013).

To evaluate the role of chromatin remodelling associated with histone modification the expression of key genes were analysed in samples collected during dormancy cycling in the SSB of the winter and summer annual Arabidopsis ecotypes Cvi and Bur. The genes selected (described above) encode enzymes involved in H2B ubiquitination (*HUB1, UBC1,* and *UBC2*) and de-ubiquitination (*OTLD1* and *UBP26*); histone acetylation (*ELO3* and *HAC1)* and deacetylation (*HD2A* and *HDA2*). The gene silencing regulators *ROS1* and *KYP/SUVH4* are included as are *CLF* and *SWN* members of the PRC2 involved in *DELAY OF GERMINATION1* (*DOG1*) regulation (Bouyer *et al*., 2012; Muller *et al*., 2012). From the results presented we propose chromatin remodelling has a dynamic role in the adjustment of dormancy status through regulation of dormancy controlling genes in response to environmental signals. To further understand this process we investigated the deposition of specific histone modifications (activating H3K4me3; repressing H3K27me3) to *DOG1* and its expression during dormancy cycling. Expression of *DOG1* responds to environmental conditions and is positively correlated with dormancy cycling in the field; it also maps to the loci with the strongest dormancy association in QTL analyses (Bentsink *et al*., 2006; Chiang *et al*., 2011; Footitt *et al*., 2011, 2013, 2014). As seeds lose dormancy H3K4me3 marks on *DOG1* chromatin decrease while H3K27me3 marks increase, as *DOG1* expression decreases (Muller *et al*., 2012). This is consistent with recently reported changes seen in germinating seeds (Molitor *et al*., 2014). Here, seeds were followed through a complete laboratory induced dormancy cycle and the pattern of activating and repressing histone modifications determined at seven positions along the *DOG1* gene. We previously suggested that *DOG1* is part of a thermal sensing mechanism that measures the passage of time (temporal sensing) with the accumulation of DOG1 protein serving to represent accumulated thermal time to regulate the depth and persistence of dormancy (Footitt *et al*., 2014). Based on the data presented we add to this by proposing that the changing proportions of H3K4me3 and H3K27me3 marks act as part of a thermal sensing mechanism in the regulation of *DOG1* transcription in line with seasonally changing soil temperature to provide another layer of regulation.

## Results

### Dormancy cycling

**Figure 1:**
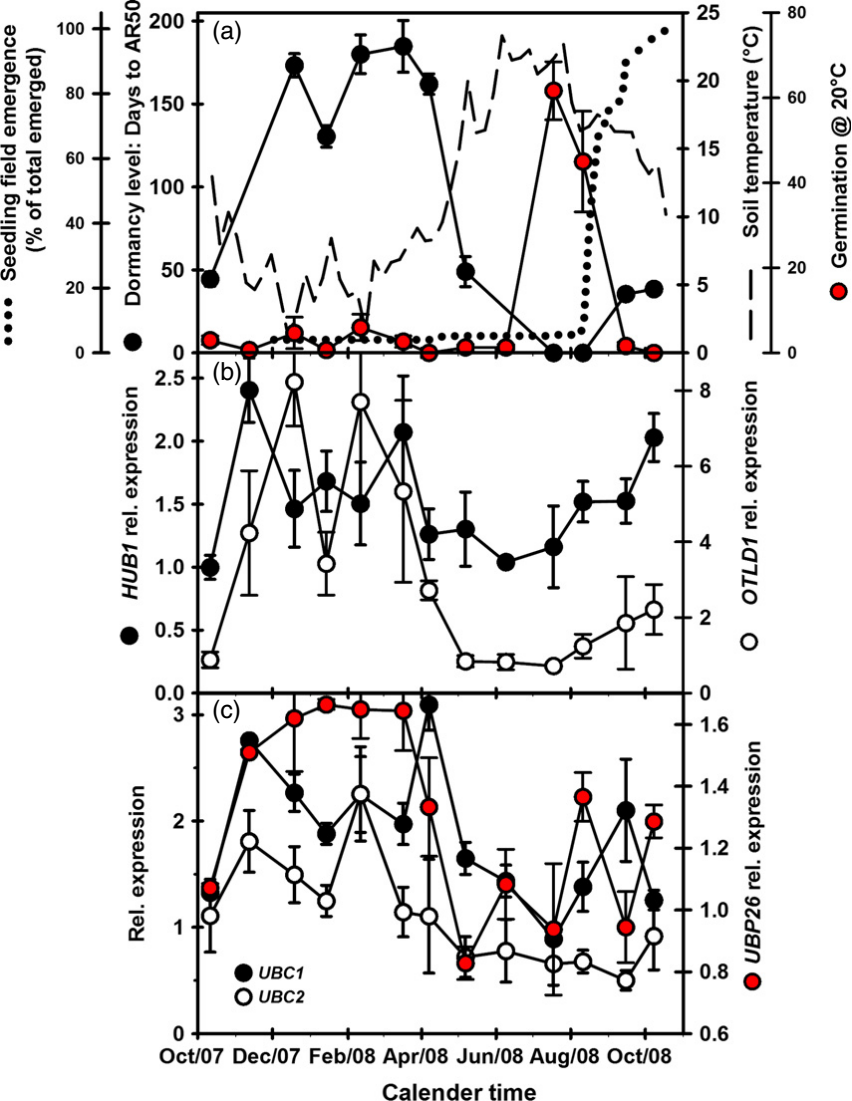
Expression of Histone2B ubiquitinating/de-ubiquitinating genes during dormancy cycling in Arabidopsis ecotype Cvi.(a) Changes in dormancy level (AR50), and soil temperature at seed depth (5 cm) over 12 months from October 2007. Mean seedling emergence following monthly soil disturbance and germination of recovered seeds incubated on water at 20°C in the light is also shown (data from Footitt *et al*., 2011).(b) Expression of *HUB1* (H2B ubiquitinating–gene activation) and *OTLD1* (H2B de-ubiquitinating–gene repression).(c) Expression of *UBC1* and *UBC2* (H2B ubiquitinating–gene activation) and *UBP26* (H2B de-ubiquitinating–gene silencing). Error bars indicate standard error of the mean, *n* = 3.

**Figure 2:**
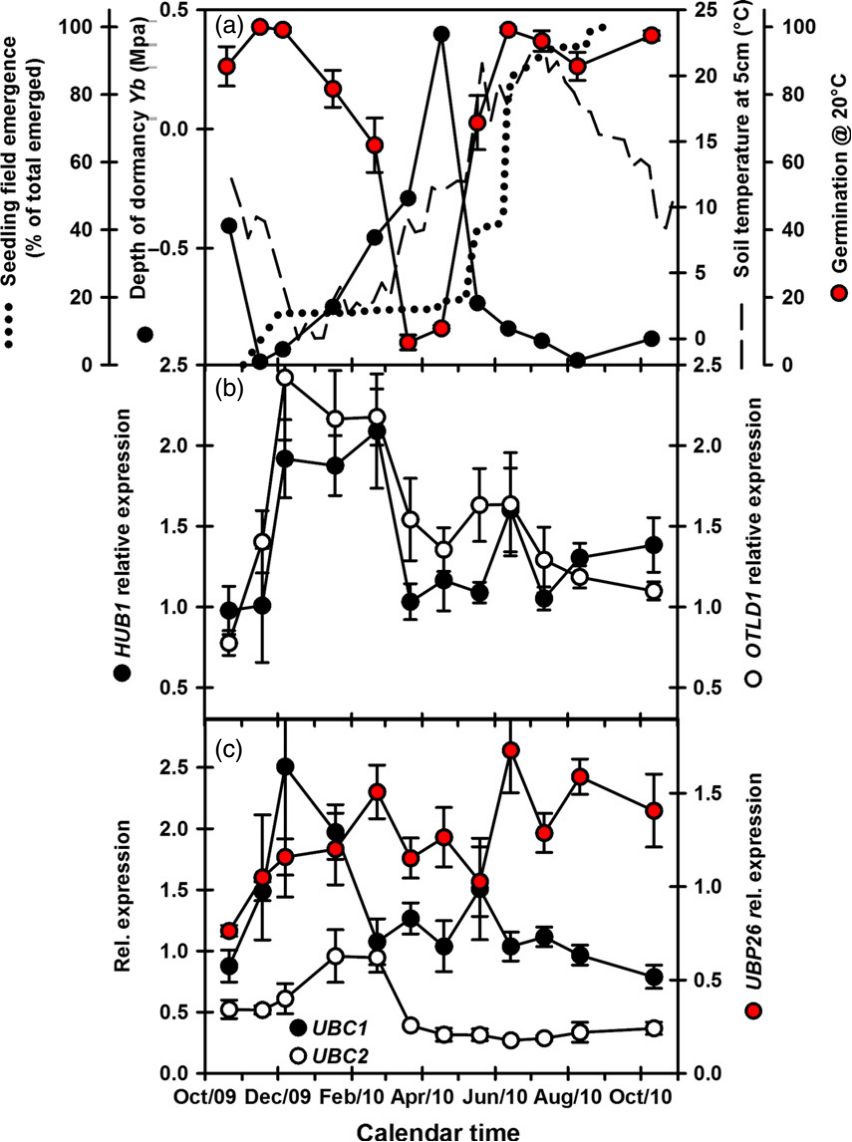
Expression of Histone2B ubiquitinating/de-ubiquitinating genes during dormancy cycling in Arabidopsis ecotype Bur.(a) Changes in dormancy level (Ψ*b*) and soil temperature measured at seed depth (5 cm) over 12 months from October 2009. Mean seedling emergence following monthly soil disturbance and germination of recovered seeds incubated on water at 20°C in the light is also shown (data from Footitt *et al*., 2013).(b) Expression of *HUB1* (H2B ubiquitinating–gene activation) and *OTLD1* (H2B de-ubiquitinating–gene repression).(c) Expression of *UBC1* and *UBC2* (H2B ubiquitinating–gene activation) and *UBP26* (H2B de-ubiquitinating–gene silencing). Error bars indicate standard error of the mean, *n* = 3.

The dormancy cycling behaviour of seeds of the Arabidopsis winter and summer annual ecotypes Cvi and Bur respectively were compared following burial in the SSB in October 2007 (Cvi) and 2009 (Bur). Dormancy in Cvi rapidly increased on burial in the field in autumn; dormancy was maximal until April after which it declined to a low point during the summer months before increasing again in autumn (Figure1a). In the summer annual ecotype Bur, upon burial dormancy initially declined before increasing from November onwards reaching a peak in April after which it declined before increasing again in autumn (Figure2a).

### Expression of histone H2B ubiquitinating/de-ubiquitinating genes

In Cvi, expression of the H2B ubiquitinating gene *HUB1* increased following seed burial and then declined in April prior to the decrease in dormancy. Expression then increased in autumn coincident with declining germination potential (Figure1a,b). In Bur, *HUB1* expression followed a similar pattern increasing following burial, reaching a peak in December then declining from February (Figure2a). In both ecotypes, increased *HUB1* expression was associated with increasing dormancy and declining germination potential (Figures1a,b and 2a,b). Expression of *OTLD1,* a H2B de-ubiquitinating gene, followed a similar pattern to *HUB1* (Figures1b and 2b), but only in Bur were the two significantly correlated (*P* < 0.01) (Table S3). However, in Cvi when dormancy declined *HUB1* (gene activation) expression remained relatively high, whereas *OTLD1* (gene repression) declined to a relatively low level. In Bur the reverse is true when dormancy is high in April. If reflected in protein levels the relative balance of gene activation via HUB1 and gene silencing via OTLD1 may influence dormancy status.

In Cvi, expression of *UBC1* and *UBC2* was positively correlated with dormancy (AR50) and *DOG1* expression and negatively correlated with soil temperature (*UBC1, P* < 0.05; *UBC2, P* < 0.01) (Figure1c; Table S3). In Bur, *UBC1* and *UBC2* expression increased on burial, but only *UBC1* had a strong pattern (Figure2c). *UBC2* expression was negatively correlated with soil temperature (*P* < 0.001) and positively correlated with *DOG1* (*P* < 0.001).

Expression of the ubiquitin-specific protease, *UBP26* in Cvi had a strong annual pattern (Figure1c) positively correlated with dormancy (AR50) (*P* < 0.05), and *DOG1* (*P* < 0.01) and negatively correlated with soil temperature (*P* < 0.01). However, in Bur, *USP26* expression increased on burial and remained at this higher level throughout the year (Figure2c). The expression patterns of genes involved in the histone 2B ubiquitination cycle in both ecotypes are consistent with published array data with the exception of the Bur *UBP26* (Table S4).

### Expression of genes encoding histone acyltransferase and deacetylating enzymes

**Figure 3:**
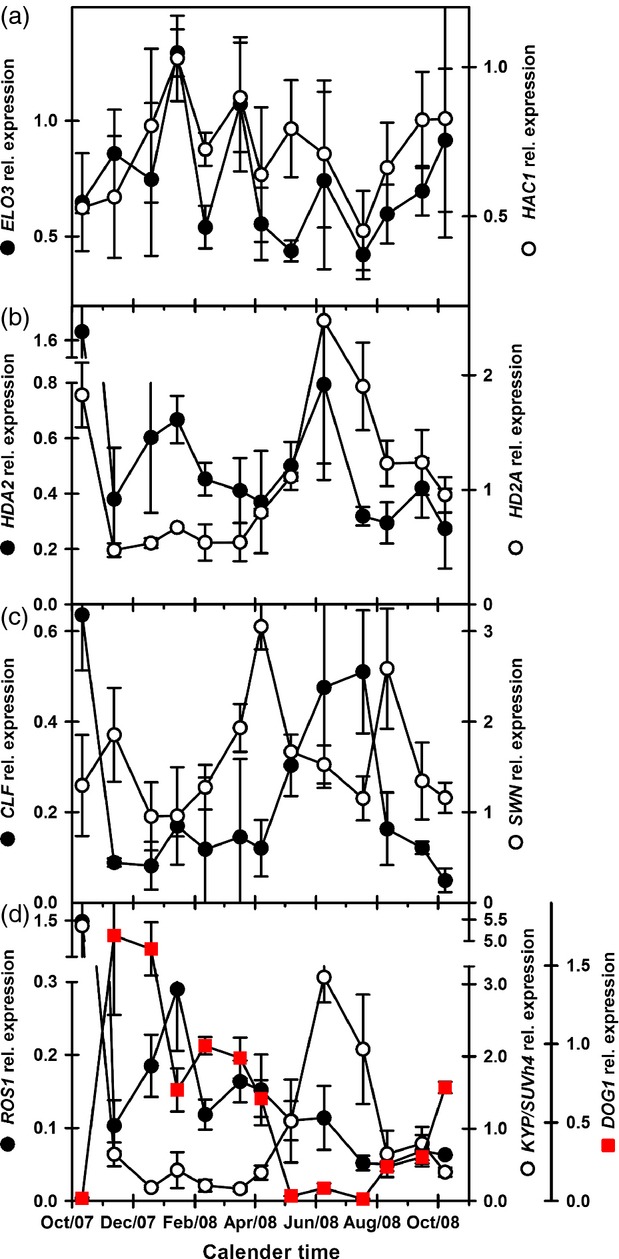
Expression of histone acetylation/deacetylating and silencing genes during dormancy cycling in Arabidopsis ecotype Cvi.(a) Expression of *ELO3* (H3K14 acetylation–gene activation) and *HAC1* (H2A, H2B, H3, and H4 acetylation–gene repression).(b) Expression of *HD2A* and *HDA2* (deacetylation–gene repression).(c) Expression of *CLF* and *SWN* (gene repression).(d) Expression of *ROS1* (suppression of gene silencing) and *KYP/SUVH4* (gene silencing) and *DOG1* (Dormancy) (*DOG1* data from Footitt *et al*., 2011). Error bars indicate standard error of the mean, *n* = 3.

**Figure 4:**
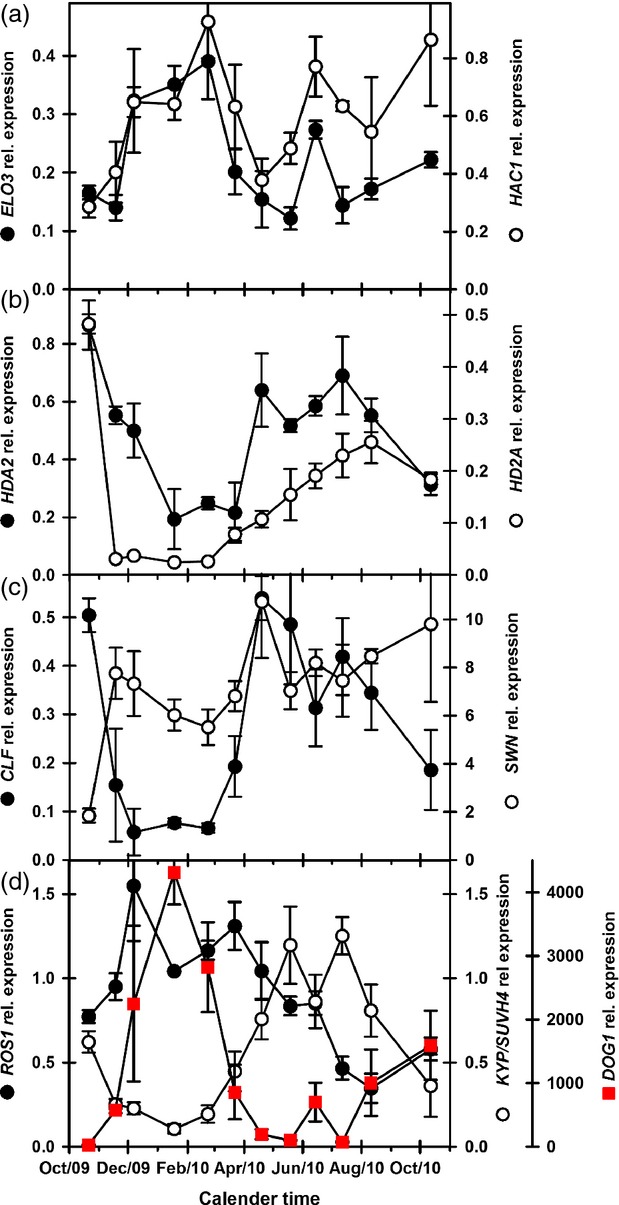
Expression of histone acetylation/deacetylation and silencing genes during dormancy cycling in Arabidopsis ecotype Bur.(a) Expression of *ELO3* (H3K14 acetylation–gene activation) and *HAC1* (H2A, H2B, H3, and H4 acetylation–gene repression).(b) Expression of *HD2A* and *HDA2* (deacetylation–gene repression).(c) Expression of *CLF* and *SWN* (gene repression).(d) Expression of *ROS1* (suppression of gene silencing) and *KYP/SUVH4* (gene silencing) and *DOG1* (Dormancy) (*DOG1* data from Footitt *et al*., 2013). Error bars indicate standard error of the mean, *n* = 3.

Numerous genes encode histone acyltransferases and deacetylating enzymes in Arabidopsis (Berr *et al*., 2011). The histone acyltransferase genes (gene activation) analysed here, (*ELO3* and *HAC1*) showed increased expression with dormancy in both ecotypes (Figures3a and 4a). *ELO3* had a second peak in summer in both ecotypes, as did *HAC1* in Bur (Figures3a and 4a). In Bur, *ELO3* expression was strongly correlated with *DOG1* expression (*P* < 0.001; Table S3). In Cvi, expression of the deacetylase genes (gene repression), *HD2A* and *HDA2,* declined on burial only for *HDA2* expression to peak as dormancy increased and again in summer coincident with the peak in *HD2A* expression and spatial sensing before both declined at the end of summer (Figure3b). In Cvi, *HD2A* expression was negatively correlated with dormancy and dormancy associated genes and positively correlated with temperature and genes associated with spatial sensing (Table S3). In Bur, expression of both genes declined on burial and increased in summer when dormancy was low and germination potential high (Figure4b). In Bur, *HD2A* and *HDA2* were positively correlated with temperature and genes related to spatial sensing (Table S3).

### Expression of the polycomb repressive complex 2 histone methyl transferases *CLF* and *SWN*

*CLF* expression had a distinct pattern in both ecotypes, following that of soil temperature but opposite to both *DOG1* expression and dormancy (Figures3c and 4c). *CLF* expression increased coincident with declining dormancy in Cvi; while in Bur, it peaked with dormancy which then declined while *CLF* levels stayed high. *CLF* expression was negatively correlated with *DOG1* expression (*P* < 0.001 (Bur) and *P* < 0.05 (Cvi); Table S3), and positively correlated with soil temperature (*P* < 0.05). In Cvi, *CLF* was negatively correlated with positive regulators of dormancy and in both ecotypes positively correlated with genes up regulated during spatial sensing. In contrast, *SWN* expression increases to a peak coincident with maximum dormancy immediately prior to dormancy loss as soil temperature increases in both ecotypes (Figures3c and 4c).

### Expression of gene silencing regulators *ROS1* and *KYP/SUVH4*

Expression of the DNA glycosylase/lyase gene, *ROS1,* which represses gene silencing, initially declined on burial, then increased during winter when dormancy increased, before declining in spring, remaining low during the spatial sensing phase of the dormancy cycle (Figure3d) with expression negatively correlated with temperature (Table S3). In Bur, *ROS1* expression increased upon burial, and then followed a pattern consistent with that in Cvi (Figure4d).

Expression of the histone methyltransferase gene *KYP⁄SUVH4,* which maintains gene silencing, had a strong pattern in both ecotypes. Expression declined rapidly on burial remaining low until late spring when it increased to a high in mid-summer before declining in autumn. Germination potential followed a similar pattern in Cvi (Figure1a). *KYP⁄SUVH4* expression was positively correlated with soil temperature (*P* < 0.01) in both ecotypes, germination in response to 50 μm GA_4+7_ (*P* < 0.05) in Cvi, and genes expressed coincident with increasing germination potential during spatial sensing in both ecotypes (Table S3) (Footitt *et al*., 2011, 2013). *KYP⁄SUVH4* was also negatively correlated with dormancy (AR50) and *MFT* (*P* < 0.01) in Cvi, and *DOG1* in both ecotypes (*P* < 0.05). In both ecotypes, *KYP⁄SUVH4* was positively correlated with *CLF* (*P* < 0.001). Although the precise timing differed, *KYP⁄SUVH4* expression increasing coincident with declining *DOG1* expression and increasing germination potential consistent with germination timing differing between the ecotypes.

### Histone modifications on *DOG1* during dormancy cycling

**Figure 5:**
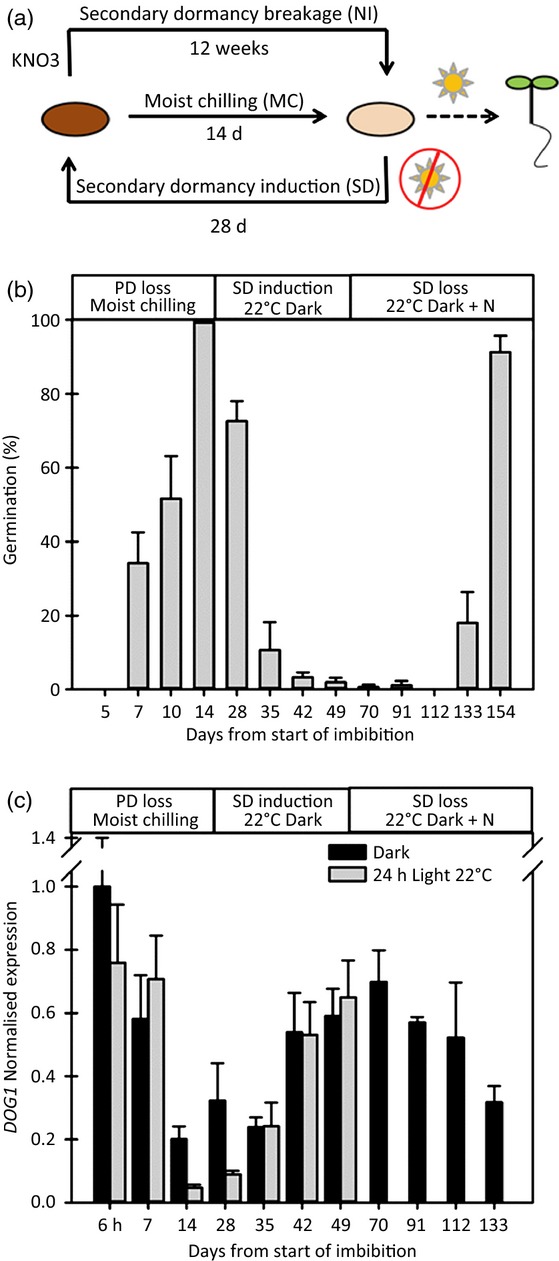
Expression of *DOG1* during dormancy cycling.(a) Schematic representation of the dormancy cycling procedure used to generate samples for *DOG1* qPCR and ChIP analysis. Primary dormancy was relieved by moist chilling (MC) at 4°C in the dark for 14 days, followed by a further 28 days at 22°C in the dark to induce secondary dormancy (SD), dormancy relief was then nitrate induced (NI) by a further 12 weeks at 22°C in the dark in the presence of 10 mmKNO_3_. As germination potential increases germination can be induced by light at 22°C.(b) Germination after 7 days in light at 22°C following transfer from conditions inducing different depths of dormancy. Samples were taken during primary dormancy (PD) loss through moist chilling (4°C per dark for 14 days), secondary dormancy (SD) was then induced by transfer to 22°C per dark, and secondary dormancy broken through exposure to 10 mm nitrate under the same conditions. Completion of germination was defined as radicle emergence.(c) *DOG1* transcript abundance (qPCR) during the same dormancy cycle. The first determination is made after 6 h during imbibition. *DOG1* transcript abundance was only recorded in the dark following transfer to nitrate. Mean values for three biological replicates are shown ± standard error (SE).

To test whether specific histone modifications were directly linked to dormancy cycling we investigated changes in histone marks on *DOG1* chromatin during dormancy cycling in the laboratory. To produce samples, Arabidopsis Cvi seeds were exposed to the artificially induced dormancy cycling under controlled conditions (Figure5a). As primary dormancy declined during moist chilling (MC) sensitivity to light increased at 22°C (Figure5b). Light sensitivity then progressively declined as secondary dormancy was induced then increased again as secondary dormancy slowly declined to a minimum (85–95% germination after 7 days in the light). *DOG1* expression followed the reverse of this (Figure5c). As dormancy declined during MC, *DOG1* expression declined rapidly on exposure of seeds to light when germination potential was >50%. As secondary dormancy increased this effect was lost. *DOG1* transcript abundance was only recorded in the dark following transfer to nitrate and subsequent loss of secondary dormancy.

**Figure 6:**
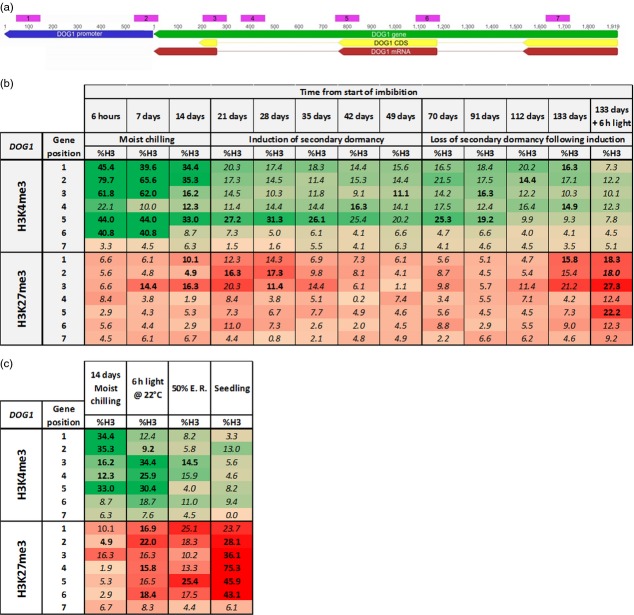
Dynamic changes in H3K4me3 and H3K27me3 marks on *DOG1* during dormancy cycling and germination.(a) Map of *DOG1* gene structure showing the seven positions targeted by primers in qPCR of DNA from the ChIP assay (Pink regions). Primers targeted to regions in which the University of California Santa Cruz Genome Browser (http://epigenomics.mcdb.ucla.edu) indicated the presence of H3K4me3 and H3K27me3 marks in seedlings.(b) Heat map showing changes (%H3, defined below) in the activating histone modification H3K4me3 (Green heat map) and the repressive modification H3K27me3 (Red heat map) at seven positions on *DOG1* during the decrease in primary dormancy in response to moist chilling, secondary dormancy induction and secondary dormancy loss as determined by chromatin immunoprecipitation followed by qPCR.(c) Heat map showing changes in the activating H3K4me3 (green heat map) and the repressive H3K27me3 (red heat map) modifications at seven positions on *DOG1* following the decrease of primary dormancy through moist chilling and subsequent removal of the final layer of dormancy by exposure to 6 h light at 22°C to induce germination, the progression of germination to 50% endosperm rupture (50% E. R.) and in seedlings. Means of three biological replicates are shown. Values were normalised to the immunoprecipitates of unmodified H3 to account for variability in histone density. Numbers in bold are significantly higher (*P* < 0.05) than background noise, which was determined by ChIP with unspecific IgG. %H3 is the fraction of modified histone H3 (as determined with the antibody against the specific methylation) within total histone H3 (as determined with an IP using an antibody against all histone H3). Colour intensity is maximum for values of 30 or greater. Standard errors and *P*-values for all means are shown in Table S5.

At the same sampling time points, two histone modifications – the activating H3K4me3, and the repressive H3K27me3 in seven regions along the *DOG1* gene were analysed by chromatin immunoprecipitation and qPCR (Figure6; Table S5). In fresh fully imbibed primary dormant seeds, H3K4me3 was present along most of the gene, with the highest concentration in the promoter and 5′-regions, and no H3K27me3 was detected (Figure6b). As dormancy declined during MC, H3K4me3 levels decreased, and towards the end of the MC period at 14 days, H3K27me3 was detected towards the 5′ end of *DOG1*. At this point, equivalent to the spatial sensing phase of the dormancy cycle in the field, dormancy had declined to a minimal level, seeds were light sensitive and germination potential was high. In this state, transfer to light to remove the final block to germination resulted in the spread of H3K27me3 over the gene in only 6 h; it then increased in concentration while H3K4me3 disappeared (Figure6c).

When secondary dormancy was induced following MC, H3K27me3 first increased slightly in the 5′ region without spreading over the whole gene, then disappeared from *DOG1* as dormancy was re-established. H3K4me3 continued to decrease for the first 7 days of secondary dormancy induction then remained stable. During the relief of secondary dormancy there was little change in the concentration of the two histone modifications or in germination potential for the first 6 weeks of nitrate treatment (91 days from the start of imbibition – DSI). With the exception of H3K4me3 at gene position 5, H3K4me3 levels remained stable through to 133 DSI. At this point H3K27me3 appeared in the 5′ region of the gene as germination potential increased. Transfer to light for 6 h at this point resulted in H3K27me3 spread over the whole gene. Concurrently H3K4me3 disappeared from the 3′ regions, and declined strongly in the 5′ region. This change on exposure to light when secondary dormancy was removed was the same as that observed when primary dormancy was removed.

## Discussion

Upon burial in autumn, seeds of both winter (Cvi) and summer (Bur) annual Arabidopsis ecotypes show rapid changes in dormancy status in response to seasonal changes, principally temperature. At the same time germination potential followed the opposite pattern (Footitt *et al*., 2011, 2013). We followed the expression of genes specifying enzymes involved in histone 2B (H2B) ubiquitination/de-ubiquitination, histone acetylation/deacetylation and related downstream histone methylation to investigate whether these environmentally-linked changes in seed dormancy were consistent with changes in chromatin structure associated with histone modifications in plants (Chinnusamy and Zhu, 2009; Berr *et al*., 2011). We also evaluated the direct impact of dormancy changing environments on the distribution of histone modifications on *DOG1* chromatin and resulting gene expression.

### Expression of genes for H2B ubiquitinating and de-ubiquitinating enzymes during seasonal dormancy cycling

The expression of *HUB1* had distinct patterns in both ecotypes. The HUB1 interacting partners UBC1 and UBC2 are redundant to each other and are orthologues of Rad6 which is highly conserved from yeast to higher eukaryotes and carries an essential ubiquitin-conjugating enzyme (UBC) domain that forms a stable conjugating complex with the yeast E3 ligase Bre1 (Du, 2012). At the protein level these ubiquitin-conjugating and E3 ligase proteins must interact to effect H2B ubiquitination, but at the transcript level, *HUB1* and *UBC1/2* expression are not strongly correlated in seeds.

Expression of the genes encoding H2B de-ubiquitinating enzymes OTLD1 and UBP26 had strong seasonal patterns. *OTLD1* was positively correlated with expression of the genes for H2B ubiquitination and *DOG1* in Bur. Highly significant correlations were seen in Cvi for *OTLD1* and *UBP26* including dormancy (AR50) and *DOG1* expression. High and simultaneous expression of genes encoding H2B ubiquitinating and de-ubiquitinating enzymes indicates a role for post-transcriptional regulation to enable seeds in the SSB to respond rapidly to changing environmental signals as a result of soil disturbance and loss of vegetative ground cover in the spatial sensing phase of the annual dormancy cycle. The greater correlation of H2B ubiquitinating and de-ubiquitinating genes with soil temperature, dormancy and *DOG1* in Cvi suggests a greater involvement of ubiquitination during deep dormancy cycling compared to the shallow dormancy cycle seen in the Bur ecotype.

### Expression of genes encoding histone acyltransferases and deacetylating enzymes during seasonal dormancy cycling

The acetylation of histones is associated with active euchromatin in a similar fashion to H2B ubiquitination. ELO3 acetylates histone 3 lysine 14 (H3K14) with H3K14ac closely correlated with the levels of H3K9ac in active promoter regions (Karmodiya *et al*., 2012). HAC1 has broad specificity acetylating histones 3 and 4 at several lysine residues (Earley *et al*., 2007). In the SSB continual maintenance of euchromatin in winter and spring is indicated by the pattern of expression of both *ELO3* and *HAC1*. A link between H2B ubiquitination and histone acetylation is suggested by the observation that *hub1 × elo3* double mutants are embryo lethal (Himanen *et al*., 2012). The increase in HAT expression seen for *ELO3* in both ecotypes and *HAC1* in Bur suggests different regions of chromatin are being activated in the temporal and spatial sensing phases of the dormancy cycle. Levels of H3K14 and H3K9 acetylation are correlated with promoter CpG content (DNA methylation sites) and gene expression levels. Weakly expressed and inactive genes have a higher H3K14ac/H3K9ac ratio, with H3K14ac more sensitive to histone deacetylases than H3K9ac indicating that inactive genes are subject to constant rounds of acetylation/deacetylation. Large numbers of these genes are subject to stimuli dependent activation indicating that H3K14 acetylation primes these genes for future activation (Karmodiya *et al*., 2012). In the SSB, ELO3 may target developmentally regulated gene expression for activation in response to environmental signals at different phases of the dormancy cycle.

The histone deacetylases of the HD2 family have contrasting roles in seeds with HD2A delaying germination in response to stress; HD2B also has a role in the down regulation of dormancy (Yano *et al*., 2006; Colville *et al*., 2011) consistent with array data for both *HD2A* and *HD2B* (Table S4). HD2A deacetylates Histone 3 Lysine 9 (H3K9) a methylation target for *KYP/SUVH4* (Berr *et al*., 2011). In the SSB, *HD2A*-mediated deacetylation during spatial sensing may enable gene silencing by *KYP/SUVH4*. Deacetylation mediated by *HDA2* appears to occur during spatial sensing in both ecotypes and during the deep dormancy phase of temporal sensing in Cvi.

### Expression of the PRC2 components *CLF* and *SWN,* and gene repression

PRC2 has a role in dormancy regulation with *DOG1* a H3K27 methylation target. PRC2 mutants have delayed germination, and maintain *DOG1* expression in seedlings (Bouyer *et al*., 2012). In the SSB, *CLF* in both ecotypes is negatively correlated with *DOG1* expression and positively with soil temperature, consistent with the increase in H3K27me3 marks on *DOG1* as dormancy declines and germination potential increases during dormancy cycling (Figure6), dormancy breaking and germination (Muller *et al*., 2012; Molitor *et al*., 2014). In contrast, *SWN* peaks with dormancy and expression may only increase further when the final layer of dormancy is removed by light on soil disturbance and germination can proceed as seen during germination where expression of *CLF* and *SWN* increased (Muller *et al*., 2012). PRC1 mutants also exhibit delays in both germination and transcriptional repression, with a delayed switch in chromatin state from H3K4me3 to H3K27me3 enrichment of *DOG1* and seed development genes (Molitor *et al*., 2014).

### *ROS1* and *KRYPTONITE* expression and gene silencing

ROS1 demethylates DNA by removal of 5-methyl cytosine to repress gene silencing (Agius *et al*., 2006): while the *KYP⁄SUVH4* histone methyltransferase mediates histone H3 lysine 9 dimethylation (H3K9me2) to permit DNA methylation resulting in gene silencing in heterochromatin (Jackson *et al*., 2004; Furner and Matzke, 2011). The high-level *ROS1* expression seen following burial that persists until spring indicates that dormancy maintenance requires repression of gene silencing. In contrast, the peak of *KYP⁄SUVH4* expression occurs in the summer when dormancy is low and germination potential high. This indicates a cohort of genes required for dormancy maintenance are maintained in a transcriptionally active state in part by ROS1, but then subjected to silencing in late spring and summer by KYP/SUVH4. Silencing may accelerate on the initiation of germination. Notably, in both ecotypes *DOG1* expression is coincident with *ROS1,* but declines and is negatively correlated with *KYP⁄SUVH4*. Crucially, *KYP⁄SUVH4* expression increased with germination potential in both ecotypes; therefore increasing earlier in Bur than in Cvi. This is consistent with *KYP/SUVH4* being a negative regulator of dormancy as *DOG1::KYP⁄SUVH4* transgenic plants have reduced *DOG1* expression (Zheng *et al*., 2012). In contrast, increased *ROS1* expression with dormancy reveals ROS1 as a positive regulator of dormancy.

### Environmental regulation of dormancy cycling: chromatin remodelling and *DOG1*

The *DOG1* gene is seed specific and strongly associated with dormancy (Bentsink *et al*., 2006; Chiang *et al*., 2011; Footitt *et al*., 2011, 2014). Key regulatory genes such as *DOG1* have bivalent promoters containing unique sets of histone modifications such as the H3K4me3 (active euchromatin) and H3K27me3 (repressed euchromatin) the ratios of which influence gene repression and silencing. Also associated with these promoters are H3K14ac and H3K9ac marks with the ratio of acetylated to unacetylated marks indicating levels of gene activation and repression. These bivalent promoters are associated with developmental regulation, with H3K27 methylation associated with genes having either low-level or tissue-specific expression (McEwen and Ferguson-Smith, 2010; Roudier *et al*., 2011; Karmodiya *et al*., 2012). These marks are deposited by histone methyl transferases. H3K4me2/3 marks are deposited by the Arabidopsis TRITHORAX (*ATX*) family while H3K27me3 marks are deposited by *CLF* and *SWN* members of the PRC2, which regulates major phase transitions in plant development (Calonje, 2014; Engelhorn *et al*., 2014). During dormancy cycling, gene activation by H3K4me3 marks is maintained by *ATX4* and gene repression by H3k27me3 marks is maintained by *CLF* and *SWN*. These genes are expression in opposite phases to each other during the dormancy cycle (Figures3 and 4; Table S4) and during dormancy breaking and germination (Muller *et al*., 2012). Consequently, during dormancy cycling the proportion of activating H3K4me3 and repressive H3K27me3 marks on *DOG1* chromatin in dormant seeds favoured H3K4me3 while the proportion of repressive H3K27me3 marks was low. As dormancy declined this proportion slowly changed but still favoured H3K4me3 until seeds were exposed to light. This indicates regulation of *DOG1* at the chromatin level is reversible until seeds are committed to germination. On exposure to light, H3K27 marks accumulated rapidly across *DOG1* chromatin while H3kme3 marks were removed as germination proceeded. At this point, stable silencing of *DOG1* may occur involving the heterochromatin marks H3K9me2 and DNA methylation at CHG*;* this requires deacetylation of H3K9ac potentially by *HD2A* (a H3K9 deacetylase; Berr *et al*., 2011) allowing formation of H3K9me2 by *KYP/SUVH4* resulting in CHG methylation (Furner and Matzke, 2011). Tellingly, *CLF, KYP* and *HD2A* expression are all significantly positively correlated with each other and soil temperature during dormancy cycling and negatively correlated with *DOG1* (Table S3).

While *DOG1* expression cycles with depth of dormancy the level of DOG1 protein only appears to change in developing seeds as dormancy is induced thereafter remaining stable with no decline even when seeds are afterripened (Nakabayashi *et al*., 2012). This is despite *DOG1* expression rapidly declining in the light as dormancy declines (Figure5) and during germination (Muller *et al*., 2012; Nakabayashi *et al*., 2012). Although the level of protein does not change it is post-translationally modified in imbibed afterripened seeds potentially rendering it inactive (Nakabayashi *et al*., 2012). DOG1 protein may only be degraded once seeds are irrevocably committed to germination when dormancy cannot be re-induced. The methylation changes we show would prevent any replacement with active DOG1 during this transition until the gene is stably silenced.

Genetic analysis indicates that several chromatin remodelling genes are involved in regulating dormancy via *DOG1. HUB1* is not required for dormancy in the presence of *DOG1;* and is not epistatic but is additive to *DOG1* with both epistatic to *KYP/SUVH4* (Liu *et al*., 2007). Additionally, mutants in the SWI-INDEPENDENT3 (SIN3)-LIKE (SNL) protein family members *SNL1* and *SNL2,* involved in histone deacetylation (gene repression) have increased H3K9/18 and H3K14 acetylation and reduced dormancy. *HUB1* and *SNL* mutants have decreased expression of *DOG1* and other dormancy associated genes and increased expression of genes associated with germination potential, While the *snl1 × hub1* mutant had lower dormancy than single mutants demonstrating the histone ubiquitination and deacetylation pathways are independent (Wang *et al*., 2013). In contrast, the *KYP/SUVH4* mutant has increased expression of *DOG1* and dormancy associated genes (Liu *et al*., 2007; Zheng *et al*., 2012; Wang *et al*., 2013). This shows that histone acetylation/deacetylation and H2B ubiquitination status independently regulate histone methylation marks via multiple histone modifications and potentially target of different cohorts of genes to regulate dormancy and germination potential.

### Histone modifications to *DOG1* may act as a thermal switching mechanism

The distribution and concentration of H3K4me3 (activating) and H3K27me3 (repressing) marks changed constantly on *DOG1* along with its expression during dormancy cycling in the laboratory. The level of H3K4me3 marks was relatively stable and retained in the promoter region but decreased in seeds with low dormancy on exposure to light. *DOG1* expression did not decrease on exposure of highly dormant seeds to light. Repressive H3K27me3 marks formed along the gene as dormancy declined, these accumulated rapidly on exposure to light. This more extreme change indicates that, at least on *DOG1,* it is the placing of H3K27me3 that is more environmentally sensitive and may be part of the thermal sensing mechanism that dominates the temporal sensing phase of the dormancy cycle. This allows regulation of DOG1 function at both the transcriptional as well as the post-translational level. Additionally, the impact of light on these marks demonstrates the role of light in removing a final layer of dormancy during spatial sensing (Footitt *et al*., 2013).

### A schematic model for the modulation of dormancy via chromatin remodelling

**Figure 7:**
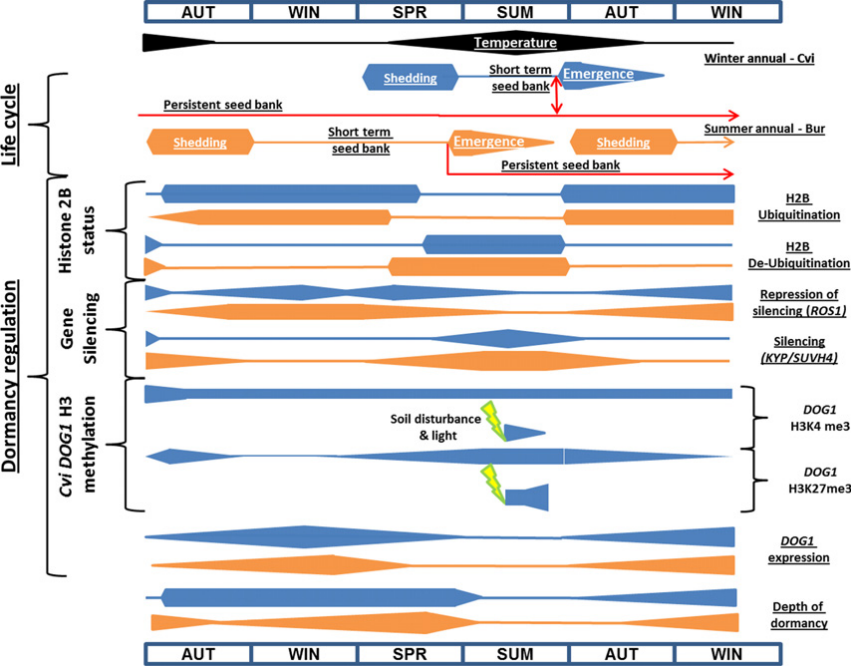
Seasonal regulation of dormancy cycling by chromatin remodelling via modulation of *DOG1* expression. The schematic incorporates data from Footitt *et al*., 2011, 2013 showing changes in soil temperature, *DOG1* expression and dormancy to illustrate how chromatin remodelling relates to *DOG1* expression during the annual dormancy cycle in the soil seed bank. The nature of chromatin remodelling is suggested by the changing expression patterns of key genes described in the text and the activating and repressive histone marks on *DOG1* chromatin. Blue and orange bars indicate Cvi and Bur ecotypes respectively. The height of each bar indicates the amplitude of the response across the seasons and yellow flashes represent exposure to light. Temperature represents the annual fluctuation in soil temperature at seed depth. Seed shedding and emergence timings are based on field observations.

As soil temperature falls in autumn, dormancy increases in response to increasing *DOG1* expression; this is amplified by H2B ubiquitination resulting in H3K4 and K3K79 tri-methylation (Figure7). At this point H2B ubiquitination is favoured over de-ubiquitination potentially by post-translational regulation of OTLD1 and UBP26. Concurrently *ROS1* (and demethylation of H3K9) removes silencing. As soil temperature increases in late spring early summer dormancy rapidly declines and H2B is de-ubiquitinated by OTLD1 and UBP26. In relation to *DOG1,* this enables H3K4 demethylation and H3K27 tri-methylation (repression). However, *DOG1* H3K4me3 marks are not removed until seeds are exposed to light, but repressive H3K27me3 marks accumulate on *DOG1* chromatin in seeds in the SSB and on soil disturbance and exposure to light this accumulation is amplified. The ability to express *DOG1* is retained until the seed is committed to germination; thereafter expression is repressed. In addition, deacetylation of H3K14 and H3K9 in a cohort of dormancy related genes enables silencing via *KYP/SUVH4* mediated histone methylation of H3K9me2. These events fit with our previous proposal that *DOG1* is part of a temporal thermal sensing mechanism with the accumulation of DOG1 serving to represent accumulated thermal time to regulate the depth and persistence of dormancy (Footitt *et al*., 2014). Here we also propose that chromatin remodelling contributes to the regulation of *DOG1* expression with *DOG1* H3K4me3 and H3K27me3 marks acting as part of this mechanism to orchestrate *DOG1* expression before it is finally silenced on the initiation of germination during spatial sensing. In late spring this would reduce DOG1 protein accumulation, with dormancy increasingly dependent only upon DOG1 protein persistence. Other changes linked to chromatin remodelling are shown in Figure S1.

In conclusion, the results presented are consistent with the dynamics of chromatin remodelling acting as a controlling gateway to the induction of changes in dormancy status. Once initiated, the expression of key genes such as *DOG1* is regulated by the maintenance of active euchromatin in these developmentally and environmentally sensitive regions of the genome. Therefore, seeds appear to respond and adapt to a changing environment by controlling gene expression at the genome level in part via changes in chromatin structure associated with histone modification. Subtle differences between ecotypes in this response may alter the timing of sensitivity to spatial environmental signals in the SSB resulting in germination and seedling emergence at the optimum time in their preferred climate space.

## Experimental Procedures

### Dormancy cycling in the field

Seeds were produced in a temperature controlled glasshouse. Mature seeds were harvested by hand threshing and equilibrated at 55% relative humidity at 20°C for 7 days to produce an equilibrium moisture content of 6–10% on a dry-weight basis. Seeds were stored at −80°C in sealed tubes. Seeds were dispersed in soda lime Ballotini balls, then placed in nylon mesh bags and buried in the field at a depth of 5 cm, before being recovered in the dark and processed as described previously (Footitt *et al*., 2011, 2013; details of seed burial, seedling emergence, germination tests, base water potential determination and subsequent gene expression analysis are described in Appendix S1 and in Table S1).

### Dormancy cycling and chromatin analysis

For laboratory studies of chromatin modification and gene expression, seeds were produced from Arabidopsis Cvi plants grown on soil in long day conditions (16 h light/8 h dark) at 22°C. Seeds were harvested at maturity, and stored at −20°C. Native chromatin immunoprecipitation and gene expression analysis were performed with modifications from those described previously (Muller *et al*., 2012) (complete details are described in Appendix S1 and in Table S2).

## Supporting Information

**Figure S1.** Seasonal regulation of dormancy cycling by chromatin remodelling via modulation of *DOG1* expression.

**Table S1.** Primers used for QPCR of field samples.

**Table S2A.** Primers used in the *DOG1*QPCR and ChIP analysis of laboratory samples.

**Table S2B.** The *DOG1*GENE and promoter (600 bp upstream).

**Table S3.** Linear correlation coefficients between environmental, physiological and molecular parameters for seeds recovered from field soils.

**Table S4.** Expression heat maps of genes involved in chromatin remodelling during dormancy cycling.

**Table S5A.** Changes in H3K4 and K3K27 methylation marks on DOG1 during dormancy relief and germination.

**Table S5B.** Changes in H3K4 and K3K27 methylation marks on DOG1 during induction of secondary dormancy.

**Table S5C.** Changes in H3K4 and K3K27 methylation marks on DOG1 during the relief of secondary dormancy.

**Appendix S1.** Experimental procedures.

## References

[b1] Agius F, Kapoor A, Zhu JK (2006). Role of the Arabidopsis DNA glycosylase/lyase ROS1 in active DNA demethylation. Proc. Natl Acad. Sci. USA.

[b2] Baskin CC, Baskin JM (2006). The natural history of soil seed banks of arable land. Weed Sci.

[b3] Bentsink L, Jowett J, Hanhart CJ, Koornneef M (2006). Cloning of DOG1, a quantitative trait locus controlling seed dormancy in Arabidopsis. Proc. Natl Acad. Sci. USA.

[b4] Berr A, Shafiq S, Shen WH (2011). Histone modifications in transcriptional activation during plant development. Biochim. Biophys. Acta.

[b5] Bouyer D, Roudier F, Heese M (2012). Polycomb repressive complex 2 controls the embryo-to-seedling phase transition. PLoS Genet.

[b6] Cadman CSC, Toorop PE, Hilhorst HWM, Finch-Savage WE (2006). Gene expression profiles of Arabidopsis Cvi seeds during dormancy cycling indicate a common underlying dormancy control mechanism. Plant J.

[b7] Calonje M (2014). PRC1 marks the difference in plant PcG repression. Mol. Plant.

[b8] Cao Y, Dai Y, Cui SJ, Ma LG (2008). Histone H2B Monoubiquitination in the chromatin of FLOWERING LOCUS C regulates flowering time in Arabidopsis. Plant Cell.

[b9] Chiang GCK, Bartsch M, Barua D, Nakabayashi K, Debieu M, Kronholm I, Koornneef M, Soppe WJJ, Donohue K, de Meaux J (2011). DOG1 expression is predicted by the seed-maturation environment and contributes to geographical variation in germination in *Arabidopsis thaliana*. Mol. Ecol.

[b10] Chinnusamy V, Zhu JK (2009). Epigenetic regulation of stress responses in plants. Curr. Opin. Plant Biol.

[b11] Colville A, Alhattab R, Hu M, Labbe H, Xing T, Miki B (2011). Role of HD2 genes in seed germination and early seedling growth in Arabidopsis. Plant Cell Rep.

[b13] Du HN (2012). Transcription, DNA damage and beyond: the roles of histone ubiquitination and deubiquitination. Curr. Protein Pept. Sci.

[b14] Earley KW, Shook MS, Brower-Toland B, Hicks L, Pikaard CS (2007). In vitro specificities of Arabidopsis co-activator histone acetyltransferases: implications for histone hyperacetylation in gene activation. Plant J.

[b15] Engelhorn J, Blanvillain R, Carles CC (2014). Gene activation and cell fate control in plants: a chromatin perspective. Cell. Mol. Life Sci.

[b16] Evans MEK, Dennehy JJ (2005). Germ banking: bet-hedging and variable release from egg and seed dormancy. Q. Rev. Biol.

[b17] Finch-Savage WE, Footitt S (2012). To germinate or not to germinate: a question of dormancy relief not germination stimulation. Seed Sci. Res.

[b18] Finch-Savage WE, Leubner-Metzger G (2006). Seed dormancy and the control of germination. New Phytol.

[b19] Finch-Savage WE, Cadman CSC, Toorop PE, Lynn JR, Hilhorst HWM (2007). Seed dormancy release in Arabidopsis Cvi by dry after-ripening, low temperature, nitrate and light shows common quantitative patterns of gene expression directed by environmentally specific sensing. Plant J.

[b20] Footitt S, Douterelo-Soler I, Clay H, Finch-Savage WE (2011). Dormancy cycling in Arabidopsis seeds is controlled by seasonally distinct hormone-signaling pathways. Proc. Natl Acad. Sci. USA.

[b21] Footitt S, Huang ZY, Clay HA, Mead A, Finch-Savage WE (2013). Temperature, light and nitrate sensing coordinate Arabidopsis seed dormancy cycling, resulting in winter and summer annual phenotypes. Plant J.

[b22] Footitt S, Clay HA, Dent K, Finch-Savage WE (2014). Environment sensing in spring-dispersed seeds of a winter annual Arabidopsis influences the regulation of dormancy to align germination potential with seasonal changes. New Phytol.

[b23] Furner IJ, Matzke M (2011). Methylation and demethylation of the Arabidopsis genome. Curr. Opin. Plant Biol.

[b24] Graeber K, Nakabayashi K, Miatton E, Leubner-Metzger G, Soppe WJJ (2012). Molecular mechanisms of seed dormancy. Plant Cell Environ.

[b25] Himanen K, Woloszynska M, Boccardi TM, Groeve S, Nelissen H, Bruno L, Vuylsteke M, Lijsebettens M (2012). Histone H2B monoubiquitination is required to reach maximal transcript levels of circadian clock genes in Arabidopsis. Plant J.

[b26] Jackson JP, Lindroth AM, Cao X, Jacobsen SE (2002). Control of CpNpG DNA methylation by the KRYPTONITE histone H3 methyltransferase. Nature.

[b27] Jackson JP, Johnson L, Jasencakova Z, Zhang X, PerezBurgos L, Singh PB, Cheng XD, Schubert I, Jenuwein T, Jacobsen SE (2004). Dimethylation of histone H3 lysine 9 is a critical mark for DNA methylation and gene silencing in *Arabidopsis thaliana*. Chromosoma.

[b28] Karmodiya K, Krebs AR, Oulad-Abdelghani M, Kimura H, Tora L (2012). H3K9 and H3K14 acetylation co-occur at many gene regulatory elements, while H3K14ac marks a subset of inactive inducible promoters in mouse embryonic stem cells. BMC Genom.

[b29] Krichevsky A, Zaltsman A, Lacroix B, Citovsky V (2011). Involvement of KDM1C histone demethylase-OTLD1 otubain-like histone deubiquitinase complexes in plant gene repression. Proc. Natl Acad. Sci. USA.

[b30] Kucera B, Cohn MA, Leubner-Metzger G (2005). Plant hormone interactions during seed dormancy release and germination. Seed Sci. Res.

[b31] Liu Y, Koornneef M, Soppe WJJ (2007). The absence of histone H2B monoubiquitination in the Arabidopsis hub1 (rdo4) mutant reveals a role for chromatin remodeling in seed dormancy. Plant Cell.

[b32] McEwen KR, Ferguson-Smith AC (2010). Distinguishing epigenetic marks of developmental and imprinting regulation. Epigenetics Chromatin.

[b33] Molitor AM, Bu Z, Yu Y, Shen W-H (2014). Arabidopsis AL PHD-PRC1 complexes promote seed germination through H3K4me3-to-H3K27me3 chromatin state switch in repression of seed developmental genes. PLoS Genet.

[b34] Muller K, Bouyer D, Schnittger A, Kermode AR (2012). Evolutionarily conserved histone methylation dynamics during seed life-cycle transitions. PLoS One.

[b35] Nakabayashi K, Bartscha M, Xianga Y, Miattona E, Pellengahra S, Yanob R, Seob M, Soppe WJJ (2012). The time required for dormancy release in Arabidopsis is determined by DELAY OF GERMINATION1 protein levels in freshly harvested seeds. Plant Cell.

[b36] Nambara E, Okamoto M, Tatematsu K, Yano R, Seo M, Kamiya Y (2010). Abscisic acid and the control of seed dormancy and germination. Seed Sci. Res.

[b37] Pons TL (1989). Breaking of seed dormancy by nitrate as a gap detection mechanism. Ann. Bot.

[b38] Roudier F, Ahmed I, Berard C (2011). Integrative epigenomic mapping defines four main chromatin states in Arabidopsis. EMBO J.

[b39] Saatkamp A, Affre L, Baumberger T, Dumas PJ, Gasmi A, Gachet S, Arene F (2011a). Soil depth detection by seeds and diurnally fluctuating temperatures: different dynamics in 10 annual plants. Plant Soil.

[b40] Saatkamp A, Affre L, Dutoit T, Poschlod P (2011b). Germination traits explain soil seed persistence across species: the case of Mediterranean annual plants in cereal fields. Ann. Bot.

[b41] Sridhar VV, Kapoor A, Zhang KL, Zhu JJ, Zhou T, Hasegawa PM, Bressan RA, Zhu JK (2007). Control of DNA methylation and heterochromatic silencing by histone H2B deubiquitination. Nature.

[b42] Sun ZW, Allis CD (2002). Ubiquitination of histone H2B regulates H3 methylation and gene silencing in yeast. Nature.

[b43] Walck JL, Hidayati SN, Dixon KW, Thompson K, Poschlod P (2011). Climate change and plant regeneration from seed. Glob. Change Biol.

[b44] Wang Z, Cao H, Sun YZ (2013). Arabidopsis paired amphipathic helix proteins SNL1 and SNL2 redundantly regulate primary seed dormancy via abscisic acid-ethylene antagonism mediated by histone deacetylation. Plant Cell.

[b45] Wright DE, Wang CY, Kao CF (2011). Flickin’ the ubiquitin switch: the role of H2B ubiquitylation in development. Epigenetics.

[b46] Yang C, Bratzel F, Hohmann N, Koch M, Turck F, Calonje M (2013). VAL- and AtBMI1-Mediated H2Aub initiate the switch from embryonic to postgerminative growth in Arabidopsis. Curr. Biol.

[b47] Yano A, Kodama Y, Koike A, Shinya T, Kim HJ, Matsumoto M, Ogita S, Wada Y, Ohad N, Sano H (2006). Interaction between methyl CpG-binding protein and Ran GTPase during cell division in tobacco cultured cells. Ann. Bot.

[b48] Yano R, Takebayashi Y, Nambara E, Kamiya Y, Seo M (2013). Combining association mapping and transcriptomics identify HD2B histone deacetylase as a genetic factor associated with seed dormancy in *Arabidopsis thaliana*. Plant J.

[b49] Zheng J, Chen FY, Wang Z, Cao H, Li XY, Deng X, Soppe WJJ, Li Y, Liu YX (2012). A novel role for histone methyltransferase KYP/SUVH4 in the control of Arabidopsis primary seed dormancy. New Phytol.

