# Supplementary figures and images for: Seed dormancy cycling in Arabidopsis: chromatin remodelling and regulation of DOG1 in response to seasonal environmental signals
Source: Plant J. 2014 Dec 26;81(3):413–25. doi: 10.1111/tpj.12735 (PMC4671266; doi:10.1111/tpj.12735)

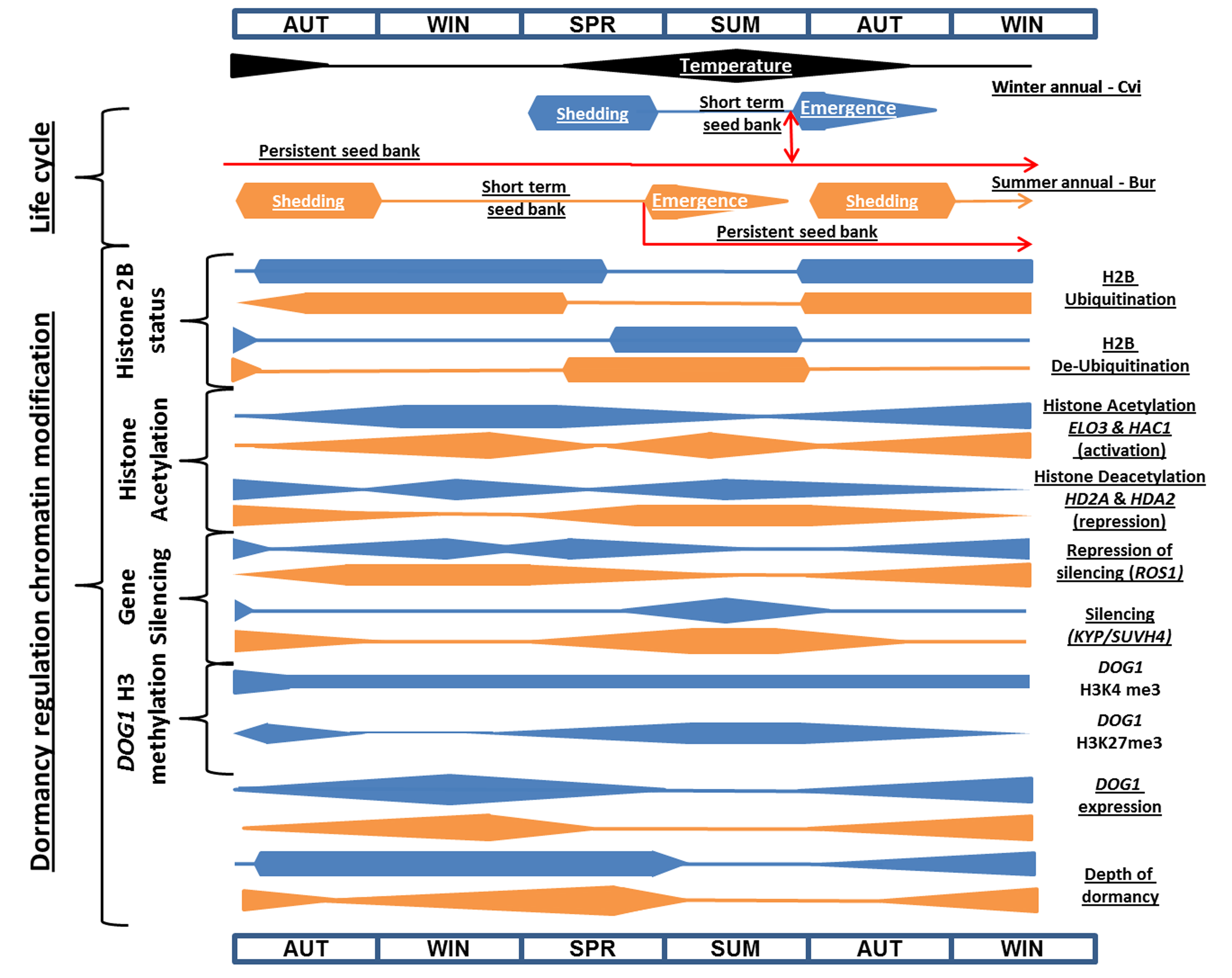

Supplement: Supplementary file 1 [file tpj0081-0413-sd1.tif]
