# Supplementary material for: Seed dormancy cycling in Arabidopsis: chromatin remodelling and regulation of DOG1 in response to seasonal environmental signals
Source: Plant J. 2014 Dec 26;81(3):413–25. doi: 10.1111/tpj.12735 (PMC4671266; doi:10.1111/tpj.12735)
Supplement: Supplementary file 2 [file tpj0081-0413-sd2.doc]

**Table S1. Primers used for QPCR of field samples.**

| Gene |  | Forward primer | Reverse primer |
| --- | --- | --- | --- |
| At4g34270 | *TIP41-like* | GTGAAAACTGTTGGAGAGAAGCAA | TCAACTGGATACCCTTTCGCA |
| At4g12590 |  | GAGATGAAAATGCCATTGATGAC | GCACCCAGACTCTTTGATG |
| At2g44950 | *HUB1* | GAGAGGGATGACTATAACATCAAGC | GCAGAGTATCTTGCATCTGCCTA |
| At1g14400 | *UBC1* | GCTATACTTACCTCCATCCAGTCC | GCTGTACATCCGAGCAGCTT |
| At2g02760 | *UBC2* | CTTTTACCTTTTCGCACAACAA | CCTCTTGAAATCCCTCATCAA |
| At3g49600 | *UBP26* | GAGACAAAGACCAGACGCTGT | ACATCATTAGCCTCGTGAATTTT |
| At2g27350 | *OTLD1* | TCTCGAATCGGTATTCTCAGC | CAAATTCTCCTCTTTTCCCGATA |
| At5g13960 | *KYP/SUVH4* | ACCGACTGAAACGATTGGAG | CGTCCAGCAACAAAGTTGAC |
| At2g3649 | *ROS1* | GGACAGAACACCGAGTTTACG | TCATCAGGTTCTCTCTTTTCCAA |
| At5g50320 | *ELO3* | TACCAGCATGCGAGCTATTC | CCGCTTCAGCTGATCTATCC |
| At1g79000 | *HAC1* | GGCTATGCGAATCAGGATTTT | TCCATATATTTAGCCTTTGATGCTT |
| At3g44750 | *HD2A* | CCACAAGGCTATTCTGAGGAA | CTACAGCCTTGGCAGCATT |
| At5g26040 | *HDA2* | AGTGCCACTGTCGCTAGGAT | GTCCCACCAACCTGCTTTC |
| At2g23380 | *CLF* (BUR) | GCAGAGAGCTCCCTTCTCAA | AGCCGGCAATCAAATACAA |
| At2g23380 | *CLF* (CVI) | GTTCGATATCAATGGAAATATGGTT | CTTTGCCTCTCCTACGTAGAAATC |
| At4g02020 | *SWN* | TGCAGAAATTGCTGGGTTAGT | GAAGTCTCATGTTTCCGCATT |
