# Supplementary material for: Seed dormancy cycling in Arabidopsis: chromatin remodelling and regulation of DOG1 in response to seasonal environmental signals
Source: Plant J. 2014 Dec 26;81(3):413–25. doi: 10.1111/tpj.12735 (PMC4671266; doi:10.1111/tpj.12735)
Supplement: Supplementary file 3 [file tpj0081-0413-sd3.doc]

**Table S2A. Primers used in the *DOG1* QPCR and ChiP analysis of laboratory** samples.

| Gene |  | Forward primer | Reverse primer |
| --- | --- | --- | --- |
| At5g45830 | *DOG1* | GGCTCGTTTATTGGTCGAGGCCG | TCCCAGCGAGCAAGAAATCCGC  GCGGATTTCTTGCTCGCTGGGA*  *reverse complement |
| At2g20000 |  | GTATAGCTCCACCACCACTT | TCTTCTAGGTGCTTGAAGAGT |
| At1g17210 |  | CTGCTTCATATGAATCACGAG | TCAACACTATCTGCACGTTGT |

| **Primers for Chip analysis at seven position on *DOG1*** | | Reverse complement |
| --- | --- | --- |
| *DOG1-1_F* | TGGAACAACAACTCGCACTC |  |
| *DOG1-1_R* | GTGCTTTCCGAGGAAATAAAAG | CTTTTATTTCCTCGGAAAGCAC |
| *DOG1-2_F* | CAAACACGCAAACCAAAAGAG |  |
| *DOG1-2_R* | GCTTGTTCGATGTTCTTTGATG | CATCAAAGAACATCGAACAAGC |
| *DOG1-3_F* | GTGACTCAGTTTCTCCGCAA |  |
| *DOG1-3_R* | GTTCCACGTGGGTGCATAAT | ATTATGCACCCACGTGGAAC |
| *DOG1-4_F* | AACATCGACGGCTACGAATC |  |
| *DOG1-4_R* | GCACCGTACTGACTACCGAAC | GTTCGGTAGTCAGTACGGTGC |
| *DOG1-5_F* | GTTTGGTTTGACAGGTGGTG |  |
| *DOG1-5_R* | TGCGTCTTCTTGTAGGCTTG | CAAGCCTACAAGAAGACGCA |
| *DOG1-6_F* | TGCATGAGTGGGGAACTATG |  |
| *DOG1-6_R* | ACGACTACTTTCCTTCCTCTCC | GGAGAGGAAGGAAAGTAGTCGT |
| *DOG1-7_F* | TCACGTCGTGGCATTTTG |  |
| *DOG1-7_R* | TCGAGACGAGATCATGTTGC | GCAACATGATCTCGTCTCGA |

Primers highlighted or in colour are shown on the *DOG1* gene sequence below
