# Supplementary material for: Seed dormancy cycling in Arabidopsis: chromatin remodelling and regulation of DOG1 in response to seasonal environmental signals
Source: Plant J. 2014 Dec 26;81(3):413–25. doi: 10.1111/tpj.12735 (PMC4671266; doi:10.1111/tpj.12735)
Supplement: Supplementary file 4 [file tpj0081-0413-sd4.docx]

**Table S2B. The *DOG1* GENE and promoter (600 bp upstream)**

Introns in italic. Primers in colour and bold.

TAATGAAGAAGAAGAAAATACAACCAAATTTACACAAAAATAATGGCAAATTAAACTATTT**TGGAACAACAACTCGCACTC**TCTATTAGTCCTCCACGTAATGCATGCATGCGTTATTT**CTTTTATTTCCTCGGAAAGCAC**GTACTAATCTTCCAACTTTCAATACTAATACTCGTTTTAATATGTGTCCAAGTAAACATTTTCTGTCCAAGTTCATTTTCAACTTTTGAGTTGTCAATGTTTCTATTTTTCCTCTTTATTTTTGACATTTGTCATTGTTTCCCTATATTAAATTTAAACGATAATAATTTGGTTCGGTTAATATAACGCTACTAAATATATATTTAGAAGAAAATTTAATTGTTTAATCATAATTCAGTTTATTCGACATTTTTATCCATAAGTATTATAATGTATAGTATTATTTTTAGAGATAATCATTAATCAGAGAATAAAATATAATTCTGGTCCATTTAACTTTTAACTATATATAAACCCAATGAAATGAAAACAAAAACACACAAAACA**CAAACACGCAAACCAAAAGAG**ATCAAACCAATAATCAAAATACTCCTCATTATATCAGAAAAAAAAAAAAAA

**ATG**GGATCTT**CATCAAAGAACATCGAACAAGC**TCAAGATTCTTATCTCGAGTGGATGAGTTTGCAATCTC

AACGCATCCCTGAGCTCAAACAACTCTTAGCTCAACGACGATCTCACGGTGATGAAGATAATGATAACAA

GCTTCGTAAGTTAACGGGAAAAATCATCG**GTGATTTCAAAAATTACGCCGCAA**AAAGAGCTGATCTTGCT

CACCGATGTAGCTCGAACT**ATTATGCACCCACGTGGAAC**AGTCCTTTAGAGAACGCTCTAATTTGGATGG

GTGGTTGTCGACCATCTTCTTTCTTTAGGCTCGTTTATGCTTTGTGTGGGTCACAAACTGAGATCCGTGT

GACTCAGTTTCTCCGC**AACATCGACGGCTACGAATC**TTCAG*G*T*AAGGGTTTGGACGTTTTCGGTTATTTC*

*GGTTTTGAGGAATAAAAAAATAGACTAAATTTAATTTTTTG****GTTCGGTAGTCAGTACGGTGC****GGCAAAAA*

*ATTTTATTGTCTTCCTTCCGATTTCTAAAATTTATTTATTCTGTTCATTTTGCTTATGACAAAAATAATA*

*GATTCTTAGGTTTTATATTAAGTTGGGTTCGGTTTGAATTTGGAATTTGTTCAGTTCTATATATTATACA*

*AATATATTATTATTTTTAAATAAAATTAATCATAAAAAATGATTCCGAACCATAGATCTATACCAAAATT*

*CTAATGGTTTCGGTTCGGTTGGTTCAATATG****GTTTGGTTTGACA*GGTGGTG**GCGGCGGTGCATCACTTAG

CGACTTAAGTGCGGAGCAGCTAGCTAAAATCAATGTGTTGCATGTAAAAATTATAGACGAAGAAGAGAAG

**ATGACCAAGAAAGTCTCAAGCCTACAAGAAGACGCAGCGGATATTCCCATCGCCACTGTGGCTTACGAGA**

TGGAGAATGTCGGAGAGCCTAACGTAGTGGTGGATCAAGCTCTCGACAAGCAAGAAGAAGCTATGGCTCG

TTTATTGGTCGAGGCCGATAATCTAAGGGTTGATACTTTAGCGAAGATCCTCGGGATTCTATCTCCGGTA

CAAGGAGCGGATTTCTTGCTCGCTGGGAAAAAGCTTCATCTTTCGA**TGCATGAGTGGGGAACTATG**AGAG

ATCGTCGCCGTCGTGACTGTATGGTTGACACCGAAGG*TAATGCCGGA****GGAGAGGAAGGAAAGTAGTCGT****T*

*ATTATATTAGATATGATACTATAGGTACGTACGTGTCATATTTAAAATTGCATAATAACAAGGTTTTCAA*

*TCTTGATATTATAAAGTTATATATTTGGCATATATTCTAGGGATATATAACCCGTATGTGTGTTACTTTT*

*ATTTTCTGAAGACTTATGGTAGCAAGGTGCAATGCGTTATGTTAGTTTATATTATAAATAATTAAGATTT*

*TGCTTTGTATAAGATGGATCTTTTGTTTTTTATTAAATGCAGAAATATAGTTTTATATATAATCTTATAT*

*AATAATTATTGTTTCGTTATAAGATTGTAGTTTGTAAGGATAAAAAATAACCTTTTTTTTGGTTAATGGA*

*TAAGAAATCATTTATAAGTTACATTTTGATGTTTTAAATTTTATCACTTATCGAAGATATATTTGAATTA*

*TTCTATTTTTTATAGTTTAT****TCACGTCGTGGCATTTTG****CGAAATACAGT*AATATTCGATGCATGTACAAC

TGTGAATAGTGGCCCACGCCCCACGGAGACGACAAATAATGAGAGAAAT**TGA**GATTTGTGTGTTTACGTG

GCA**GCAACATGATCTCGTCTCGA**CAAGTCAGCTAGGAGATTTTTATCCAAGATTGCATTGGATTTTTATA

TTCTTAGATTTTTCCATTATTATTTTGTGTGGTAGGGAATATTCATAAAGAAAATTGTGGAGTATCTAAA

TAAATTGTTGGAAGCATCCTTTTTTTTTC
