# Supplementary material for: Seed dormancy cycling in Arabidopsis: chromatin remodelling and regulation of DOG1 in response to seasonal environmental signals
Source: Plant J. 2014 Dec 26;81(3):413–25. doi: 10.1111/tpj.12735 (PMC4671266; doi:10.1111/tpj.12735)
Supplement: Supplementary file 10 [file tpj0081-0413-sd10.docx]

**Experimental Procedures**

**Seed production:** For field burial studies, seeds of the Arabidopsis Cvi and Bur ecotypes were produced in a heated glasshouse with supplemental lighting. Seeds were harvested at maturity and stored at -80°C (Footitt et al. 2011, Footitt et al. 2013). For laboratory studies of chromatin modification and gene expression, seeds were produced from Arabidopsis Cvi plants grown on soil in long day conditions (16 h light / 8 h dark) at 22°C. Seeds were harvested at maturity, and stored at -20°C. Biological replicates consisted of seeds pooled from five to eight plants.

**Burial and recovery from the soil seed bank:** Seeds for burial in 2007 (Cvi) and 2009 (Bur) were dispersed in Ballotini balls in nylon mesh bags (Potters Ballotini Ltd, Barnsley, UK) and buried in field plots at a depth of 5 cm in a randomised plot design. Thermistore temperature probes (Betatherm, Ireland) linked to a data logger (Delta-T Devices Ltd, Cambridge, UK) recorded soil temperature at seed depth in dummy bags. Seeds for molecular analysis were recovered from the SSB and flash frozen in liquid nitrogen in the dark using previously published procedures (Footitt et al. 2011, Footitt et al. 2013).

**Dormancy testing of recovered seeds:** Exhumed seeds were surface-sterilized in a 0.125 % sodium hypochlorite solution (household bleach: 5% sodium hypochlorite, diluted to 2.5%) for 5 min then washed three times in water. Thermodormancy at 20°C was tested as follows; 40 seeds were plated out in triplicate plates (124 x 88 x 22 mm) (Stewart Plastics Ltd, Croydon, UK). Each plate contained two sheets of Whatman 3MM chromatography paper (Scientific Laboratory Supplies Ltd, Nottingham, UK) and 8 mL of water. Plates were incubated at 20°C under continuous light and germination scored at 2-3 day intervals for up to 28 days. Germination was recorded as emergence of the radicle through the seed coat. Depth of dormancy was determined in two ways. In the deeply dormant Cvi ecotype dormancy was measured as days of dry afterripening at 20°C required for 50% germination at 20°C in the light (AR50). In the shallow dormant Bur ecotype a hydrothermal time approach was taken and dormancy evaluated as base water potential (*Ψb*) expressed as Mega Pascals (MPa) (Footitt et al. 2013).

**Seedling emergence:** Seeds of each ecotype were buried in the field on 2/11/2009 and emergence tests carried out as described previously by thoroughly stirring the soil at seed depth in each pot monthly (Footitt et al. 2011).

**Gene expression analysis in field samples:** RNA was extracted from seeds as described previously (Footitt et al. 2013). cDNA synthesis and quantitative PCR (QPCR) was performed in triplicate on each of three independent biological samples using a 1/25 dilution of cDNA. QPCR was performed on a Roche LightCycler® 480 with data analysed using LightCycler® 480 software (version 1.5; Roche Diagnostics, Burgess Hill, UK). Genes chosen for analysis were *HUB1* (At2g44950), *UBC1* (At1g14400), *UBC2* (At2g02760)*, UBP26* (At3g49600), *OTLD1* (At2g27350), *ELO3* (At5g50320), *HAC1* (At1g79000), *HD2A* (At3g44750), *HDA2* (At5g26040), *ROS1* (At2g36490), *KYP⁄SUVH4* (At5g13960), *CLF* (At2g23380) and *SWN* (At4g02020). Primer sequences are given in Table S1. Gene expression levels were determined using a cDNA dilution series for each gene of interest with normalisation against the combined geometric mean at each time point of the reference genes At4G34270 (Tip 41-like) and At4g12590. These reference genes have a low fold change in expression and coefficient of variation across 11 different hydrated dormancy states in published micro array data; they were also identified as highly stable reference genes for use in expression analysis by QPCR in Arabidopsis seeds (Cadman et al. 2006, Finch-Savage et al. 2007, Dekkers *et al.* 2012).

**Chromatin analysis:**

**Dormancy cycling, sampling and germination treatments:** Dormant Cvi seeds were cycled through dormancy after Cadman et al. (Cadman et al. 2006). Seeds were imbibed on water in the dark at 4⁰C (moist chilling) for up to 14 days to relieve primary dormancy. Seeds were then transferred to 22⁰C in the dark for up to 28 days to induce secondary dormancy. Following this treatment secondary dormancy was relieved by transferring seeds to agar plates containing 10 mM potassium nitrate at 22⁰C in the dark. Whole plates of seeds were harvested at the times indicated, and seeds from each plate used for RNA extraction, chromatin immunoprecipitation (ChIP), and germination testing. Seeds were only exposed to light when germination potential was determined as follows; three biological replicates of 50 seeds were removed from each treatment at regular intervals, and sown on 1% agar. Completion of germination was counted after 7 d of incubation at 22⁰C in long day conditions. The population was considered fully non-dormant when they germinated to 100% within the 7 d.

**Native chromatin immunoprecipitation (nChIP):** Seeds for use in nChIP were flash frozen in liquid nitrogen and stored at -80⁰C. nChIP was performed as described previously (Muller et al. 2012) with the following modification: 2 mM CaCl_2_ was added to the nuclease buffer, and the antibodies used against modified histones were rabbit multiclonal antibody C42D8 against H3K4me3 and rabbit multiclonal C36B11 against H3K27me3 (Cell Signaling Technology, Beverly, USA). Three µl of each of these antibodies were added for each of the respective immunoprecipitation reactions.

**Gene expression analysis in laboratory samples:** Seeds for RNA extraction were flash frozen in liquid nitrogen and stored at -80°C. Seeds were ground in liquid nitrogen, total RNA was extracted and 1 μg reverse transcribed as described previously (Muller et al. 2012). cDNA from three biological replicate RNA samples was used for QPCR of *DOG1* (At5g45830). qPCRs were run in 15 μl reaction volumes on an ABI7900HT machine (Applied Biosystems, Grand Island, USA) using the PerfeCTa Sybr Green Supermix with ROX (Qanta Biosciences, Gaithersburg, USA).The reaction mixture consisted of 150 ng cDNA (RNA equivalent) or 1 µl of 1:5 diluted purified DNA from the ChIP assay. Supermix (7.5 μl) and 140 nM of each primer were used, and the temperature regime was 3 min at 95°C and 40 cycles of 15 s at 95°C/1 min at 60°C. Primers were placed in the regions in which the UCSC Genome Browser (http://epigenomics.mcdb.ucla.edu, 21) indicated the presence of H3K4me3 and H3K27me3 marks in seedlings. Primers sequences are given in Table S2. Data was analysed as described elsewhere (Muller et al. 2012). Gene expression levels were normalised against the combined geometric mean at each time point of the reference genes *At1g17210* and At2g20000.

**Cadman, C.S.C., Toorop, P.E., Hilhorst, H.W.M. and Finch-Savage, W.E.** (2006) Gene expression profiles of Arabidopsis Cvi seeds during dormancy cycling indicate a common underlying dormancy control mechanism. *Plant Journal*, **46**, 805-822.

**Dekkers, B.J.W., Willems, L., Bassel, G.W., van Bolderen-Veldkamp, R.P., Ligterink, W., Hilhorst, H.W.M. and Bentsink, L.** (2012) Identification of Reference Genes for RT-qPCR Expression Analysis in Arabidopsis and Tomato Seeds. *Plant and Cell Physiology*, **53**, 28-37.

**Finch-Savage, W.E., Cadman, C.S.C., Toorop, P.E., Lynn, J.R. and Hilhorst, H.W.M.** (2007) Seed dormancy release in Arabidopsis Cvi by dry after-ripening, low temperature, nitrate and light shows common quantitative patterns of gene expression directed by environmentally specific sensing. *Plant Journal*, **51**, 60-78.

**Footitt, S., Douterelo-Soler, I., Clay, H. and Finch-Savage, W.E.** (2011) Dormancy cycling in Arabidopsis seeds is controlled by seasonally distinct hormone-signaling pathways. *Proceedings of the National Academy of Sciences of the United States of America*, **108**, 20236-20241.

**Footitt, S. and Finch-Savage, W.E.** (2011) 'Production of Seed Samples for the Effective Molecular Analysis of Dormancy Cycling in Arabidopsis In *Seed Dormancy. Methods in Molecular Biology* (Kermode, A. ed: Springer, pp. 65 - 79.

**Muller, K., Bouyer, D., Schnittger, A. and Kermode, A.R.** (2012) Evolutionarily Conserved Histone Methylation Dynamics during Seed Life-Cycle Transitions. *Plos One*, **7**, e51532.
