# Supplementary material for: Seed dormancy cycling in Arabidopsis: chromatin remodelling and regulation of DOG1 in response to seasonal environmental signals
Source: Plant J. 2014 Dec 26;81(3):413–25. doi: 10.1111/tpj.12735 (PMC4671266; doi:10.1111/tpj.12735)
Supplement: Supplementary file 11 [file tpj0081-0413-sd11.docx]

**Figure S1: Seasonal regulation of dormancy cycling by chromatin remodelling via modulation of *DOG1* expression**. The schematic summarises data from Footitt et al., 2011, 2013 to show changes in soil temperature, *DOG1* expression and dormancy. It uses these data to illustrate how chromatin remodelling relates to *DOG1* expression during the annual dormancy cycle in the soil seed bank. The nature of chromatin remodelling is suggested by the changing expression patterns of genes involved in histone 2B (H2B) ubiquitination (gene activation) and de-ubiquitination (gene repression), histone acetylation (gene activation) and de-acectylation (gene repression) and related gene silencing and the repression of gene silencing. The coordination of these responses to soil temperature represents a potential mechanism for the regulation of temporal sensing in seeds of both winter and summer annual Arabidopsis ecotypes. Subtle changes in the timing of these events such as de-ubiquitination, gene silencing and reduced *DOG1* expression are earlier in Bur than Cvi consistent with the different times of germination and seedling emergence in disturbed field soil. In the schematic, the winter annual Cvi is represented by the blue bars and the summer annual ecotype Bur by the orange bars. The height of each bar indicates the amplitude of the response across the seasons. Temperature represents the annual fluctuation in soil temperature at seed depth. Seed shedding times and emergence timing are based on field observations.

**Table S1. Primers used for QPCR of field samples.**

**Table S2A. Primers used in the *DOG1* QPCR and ChiP analysis of laboratory samples.**

**Table S2B. The *DOG1* GENE and promoter (600 bp upstream)**

Gene sequence of *DOG1.* Intron sequences are shown in italics. Primers positions are shown in colour and bold.

**Table S3: Linear correlation coefficients between environmental, physiological and molecular parameters for seeds recovered from field soils.** In order to consider only the response to seed burial data from seeds prior to burial was omitted as their response is determined by prior maternal maturation conditions. Data for genes not shown in this paper are in Footitt et al., 2011 and 2013.

**Table S4: Expression heat maps of genes involved in chromatin remodelling during dormancy cycling.** Identifiers of dormant states used in heat maps are described below. Data comparisons are from the transcriptome analysis presented in Cadman et al., (2006) and Finch-Savage et al., (2007). Heat maps generated using Heatmapper plus (<http://bar.utoronto.ca/ntools/cgi-bin/ntools_heatmapper_plus.cgi>)

**Table S5A: Changes in H3K4 and K3K27 methylation marks on DOG1 during dormancy relief and germination.** Dormancy was relieved by moist chilling at 4°C in the dark and germination induced at 22°C in the light. After 14 days moist chilling at 4°C in the dark(MC), seeds were transfered to 22°C in the light and sampled after 6 hours; at 50% endosperm rupture and radicle emegence (completion of germination)(50% ER) and in seedlings. A temperature control was also performed were dormant seeds where maintained for 14 days at 22°C in the dark and remained dormant (22°C control). Numbers in red indicate the Mock IP negative control (unspecific IgG IP) was the same or higher than the specific H3 methyaltion value and is therefore considered as background noise. ChIP data was analysed for significance using paired t-tests, P-values show the level of significance for each comparison. Numbers in bold are significantly higher than the controls at p≤0.05. (Data are mean ± standard error of the mean; n=3.)

**Table S5B: Changes in H3K4 and K3K27 methylation marks on DOG1 during induction of secondary dormancy.** Dormancy was first relieved by moist chilling 4°C in the dark for 14 days (MC) followed by the induction of secondary dormancy s at 22°C in the dark. Numbers in red indicate the Mock IP negative control (unspecific IgG IP) was the same or higher than the specific H3 methyaltion value and is therefore considered as background noise. ChIP data was analysed for significance using paired t-tests, P-values show the level of significance for each comparison. Numbers in bold are significantly higher than the controls at p≤0.05. (Data are mean ± standard error of the mean; n=3).

**Table S5C: Changes in H3K4 and K3K27 methylation marks on DOG1 during the relief of secondary dormancy.** Secondary dormancy was relieved at 22°C in the dark in the presence of 10 mM KNO3. After 163 days methylation pattern was determined after 6 hours exposure to light (germination inducing) (163 days + 6h Light). Numbers in red indicate the Mock IP negative control (unspecific IgG IP) was the same or higher than the specific H3 methyaltion value and is therefore considered as background noise. ChIP data was analysed for significance using paired t-tests, P-values show the level of significance for each comparison. Numbers in bold are significantly higher than the controls at p≤0.05. (Data are mean ± standard error of the mean; n=3).
